# Do presenting symptoms predict treatment decisions and survival in glioblastoma? Real-world data from 1458 patients in the Swedish brain tumor registry

**DOI:** 10.1093/nop/npae036

**Published:** 2024-04-23

**Authors:** Helena Bruhn, Björn Tavelin, Lena Rosenlund, Roger Henriksson

**Affiliations:** Department of Biomedical and Clinical Sciences, Linköping University, Linköping, Sweden; Clinical Research Unit, Cancercentrum, Region Vasterbotten, Umea University Hospital, Umea, Sweden; Regional Cancer Centre, Stockholm, Sweden; Department of Radiation Sciences, Oncology, Umea University Hospital, Umea, Sweden

**Keywords:** cognition, glioblastoma, prognostic factors, survival, symptoms

## Abstract

**Background:**

Glioblastoma is the most common malignant brain tumor in adults. Non-invasive clinical parameters could play a crucial role in treatment planning and serve as predictors of patient survival. Our register-based real-life study aimed to investigate the prognostic value of presenting symptoms.

**Methods:**

Data on presenting symptoms and survival, as well as known prognostic factors, were retrieved for all glioblastoma patients in Sweden registered in the Swedish Brain Tumor Registry between 2018 and 2021. The prognostic impact of different presenting symptoms was calculated using the Cox proportional hazard model.

**Results:**

Data from 1458 adults with pathologically verified IDH wild-type glioblastoma were analyzed. Median survival time was 345 days. The 2-year survival rate was 21.5%. Registered presenting symptoms were focal neurological deficits, cognitive dysfunction, headache, epilepsy, signs of raised intracranial pressure, and cranial nerve symptoms, with some patients having multiple symptoms. Patients with initial cognitive dysfunction had significantly shorter survival than patients without; 265 days (245–285) vs. 409 days (365–453; *P* < .001). The reduced survival remained after Cox regression adjusting for known prognostic factors. Patients presenting with seizures and patients with headaches had significantly longer overall survival compared to patients without these symptoms, but the difference was not retained in multivariate analysis. Patients with cognitive deficits were less likely to have radical surgery and to receive extensive anti-neoplastic nonsurgical treatment.

**Conclusions:**

This extensive real-life study reveals that initial cognitive impairment acts as an independent negative predictive factor for treatment decisions and adversely affects survival outcomes in glioblastoma patients.

Glioblastomas are the most prevalent type of malignant brain tumor in adults. The median life span is between 9 to 15 months, with very few patients reaching the 5-year survival mark.^1,2^ The cornerstones of treatment are maximum safe resection followed by temozolomide, initially in combination with radiotherapy, followed by 6 monthly cycles of treatment.^3–5^ Currently, prognostic markers such as younger age, preoperative performance status, the extent of surgical resection, and MGMT methylation status are utilized.^6,7^ Additionally, survival rates appear slightly higher in women than in men.^8^ There is a need for improved non-invasive prognostic markers to allow physicians to predict patient outcomes more accurately and assist patients in understanding their condition and treatment options.

Early symptoms of brain tumors can be varied and nonspecific and include signs of raised intracranial pressure, focal neurological deficits, cognitive impairment, and headache, often presenting in combination. New focal or generalized seizures in adults may also indicate a brain tumor.^9,10^ While patients’ symptoms are one of the most easily evaluable factors to assess, the research on the prognostic significance of different presenting symptoms has been limited to small-scale studies,^11,12^ with the notable exception of epilepsy.^13^ In Sweden, presenting symptoms in people diagnosed with a primary brain tumor are registered in the Swedish National Quality Registry for Adult Patients.^14^ This nationwide register-based study aimed to determine if presenting symptoms offer prognostic value for patients with glioblastoma in a real-life setting.

## Materials and Methods

### The Swedish Brain Tumor Registry, SBTR

The SBTR is the National Quality Registry for Primary Brain Tumors, and it covers all 6 healthcare regions in Sweden.

### Inclusion Criteria

All patients registered in the SBTR who underwent primary surgery or biopsy with a pathologically verified diagnosis of glioblastoma between January 1st, 2018, and December 31st, 2021, were identified and included. The registration of IDH mutation status started in 2018, determining the first inclusion date. The SBTR coding is done according to ICD-O/3.2. This included all cases with SNOMED codes 94 403, 94 413, and 94 423.

### Data Collection

Data from SBTR were retrieved in May 2022. For this study, data on clinical factors, such as age, sex, preoperative performance status (PPS), type of surgery, tumor size, extent of surgical resection (radical surgery, partial surgery, or biopsy), pathology (MGMT promoter methylation status and IDH1/2 mutations), anti-neoplastic treatment, survival and presenting symptom(s) were retrieved. Presenting symptoms are registered in groups: Focal neurological deficits, epilepsy, symptoms of raised intracranial pressure, headache, and cranial nerve symptoms, cognitive symptoms (including personality changes, memory deficit, inhibited psychomotor function, executive dysfunction, neglect, and depression) or no symptoms (incidental finding). Evaluation is done by the treating physician and then registered as yes/no variables. For non-surgical anti-neoplastic treatment, there is a separate form. Dates where treatment with radiotherapy (RT) and chemotherapy (CT) are started are noted. Patients having concomitant treatment have the same date for starting RT and CT. Patients receiving only CT or RT have only one date registered. The data on adjuvant chemotherapy were scarce, and hence not investigated. There is also an indicator for no non-surgical treatment (best supportive care).

### Data Analyses

Registered prognostic and clinical factors were analyzed for the whole cohort. Survival was calculated with the Kaplan–Meier method, and differences in survival between subgroups were evaluated with the log-rank test. Survival data are presented as medians with 95% CIs.

We included presenting symptoms where univariate analysis showed a difference in survival between having and not having the symptom, in a multivariate Cox proportional hazard model taking the known prognostic factors of age, tumor size, sex, preoperative performance status (PPS), the extent of surgical resection, MGMT promoter methylation status and oncological treatment into account. Significant differences in treatment received, in relation to presenting symptoms, were examined by univariate analysis. Where significance was found, a log-rank test was added. Since the cognitive status of the patient could influence treatment decisions, we did an additional Cox proportional hazard model, taking only initial symptoms and preoperatively known prognostic factors into account; “the treatment naïve model.”

This study was approved by the Ethical Review Authority, Umea, Sweden, numbers 2014-95-31 and 2020-06886. All statistical analyses were performed with IBM SPSS Statistics for Windows, Version 26.0. Armonk, NY, USA.

## Results

The registration rate in SBTR, compared to the mandatory National Cancer Registry, was 91-99.5%. This study includes all 1458 patients with pathologically verified IDH wild-type glioblastoma diagnosed and reported to the registry in 2018–2021 (Table 1). The median age of the patients was 66 years, ranging from 18 to 89 years. Forty-one percent were females. A high proportion of patients (32%) had a WHO performance status (PFS) of 0, and only 2% had a PFS of 4.

**Table 1.** Descriptive Data and Survival

Variable	*N* (%)	Median survival (days)	95% CI
Overall survival	1458(100)	345	326–364
*Age*			
<39 years	36 (2.5)	687	411–963
40–54 years	244 (16.7)	599	484–714
55–69 years	625 (42.9)	364	335–393
70-years	553 (37.9)	239	216–262
*WHO performance status*			
0–1	813 (55.8)	410	380–440
2	378 (25.9)	272	242–302
3–4	162 (11.1)	176	122–230
Missing	105 (7.2)		
*Tumour Size*			
<4 cm	562 (38.5)	378	347–409
4–6 cm	672 (46.1)	345	313–377
>6 cm	182 (12.5)	256	230–282
Missing	42 (2.9)		
*Type of surgery*			
Biopsy	489 (33.5)	189	166–212
Partial resection	474 (32.5)	364	328–400
Radical resection	493 (33.8)	514	460–568
Missing	7 (0.5)		
*MGMT promoter methylation status*			
Unmethylated	724 (49.7)	297	275–319
Methylated	528 (36.2)	443	381–505
Not tested	199 (13.6)	384	309–459
Missing	7 (0.5)		

Preoperative MRI was performed in 96% of patients. Tumors were evenly located on the left and right hemispheres. Most tumors were 4–6 cm in their largest diameter followed by smaller tumors (< 4 cm), and only a small proportion were larger than 6 cm (Table 1).

## Survival

Median survival was 345 days (326–364), and the 2-year survival was 21.5% (Table 1). In this population, there was no survival benefit for women versus men. Increasing age significantly decreased overall survival (OS; *P* < .001). Survival significantly decreased with decreasing preoperative performance status (*P* < .001). Increasing tumor size gradually and significantly decreased survival (*P* = .001). There was no difference in survival depending on the hemisphere.

**Figure 1.**
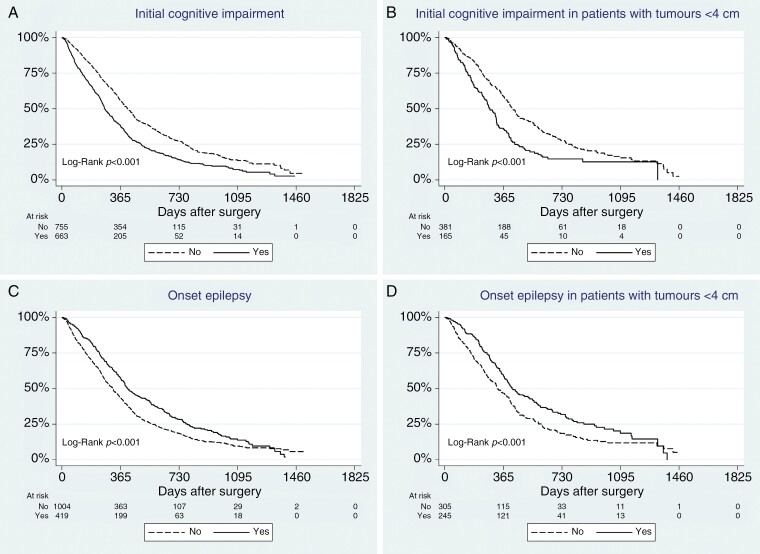
(A) Kaplan–Meyer analysis: Initial cognitive impairment. (B) Kaplan–Meyer Analysis: Initial cognitive impairment in tumors < 4 cm. (C) Kaplan–Meyer analysis: Initial epilepsy. (D) Kaplan–Meyer analysis: Initial epilepsy in tumors < 4 cm.

*MGMT-methylation status*: 36% were MGMT methylated, 50% had unmethylated MGMT and 14% of tumors were registered as unknown MGMT methylation status. There was a significant difference in survival between the MGMT methylated and MGMT unmethylated groups favoring MGMT methylated tumors, *P* < .001 (Figure 1A).

## Treatment

### Surgical treatment

 The frequency of different surgical methods was evenly distributed between radical surgery, partial surgery, and biopsy. Postoperative MRI or CT was performed after 63% of all surgeries, ie, almost all of the radical and partial surgeries. The frequency of biopsies increased with increasing age, from 22.2% among patients < 39 years to 42.2% among patients over 70 years. Radical surgeries, on the other hand, were most frequent among the youngest patients, 33.3%, for those < 39 years. compared to 29.3% among those > 70 years. Overall survival was longest among those who had undergone radical surgery and shortest among those who had only been biopsied. There were no sex differences in surgical management. The likelihood of having radical surgery did not vary between patients presenting with or without epilepsy. Of patients presenting with cognitive dysfunction, 30.6% had radical surgery, compared to 37.1% among patients with no cognitive dysfunction (*P* = .0037). The likelihood of having radical surgery was equal for patients with and without focal neurological deficits. Among patients with headaches, the frequency of biopsy was significantly lower than for patients without headaches (26.3% vs 37.5%, *P* < .001).

### Nonsurgical anti-neoplastic treatment

 At the time of data retrieval, 27% of patients had missing data on nonsurgical anti-neoplastic treatment or best supportive care in the registry.

For patients whose data were reported in the registry (*N* = 1068), 64% received concomitant radiochemotherapy (CRT). Thirteen percent had radiotherapy (RT) alone, and 12% had only chemotherapy (CT). Ten percent had no non-surgical anti-neoplastic treatment and received the best supportive care (BSC). Patients with epilepsy at debut had the highest percentage of CRT, 74% and there was a significant difference compared to patients without epilepsy (*P* < .001), where 60% had CRT. Among patients presenting with *t* cognitive dysfunction, 57% received CRT, compared to 70% for the group without cognitive symptoms, *P* < .001. For patients with/without headaches and patients with/without focal neurological deficits, there were no differences, and the frequency of CRT was 64% and 62%, respectively.

Having epilepsy reduced the likelihood of having BSC to 5%, compared to 12% amongst patients with no epilepsy. Fourteen percent of patients with cognitive dysfunction received BSC versus 7% for those without. Presenting with focal neurological deficits or headaches did not affect the likelihood of having BSC.

## Presenting Symptoms

At the time of discovery, focal neurological deficits were present in 64% of cases, 46% had cognitive dysfunction and 36% had headaches. Epilepsy at presentation was described in 29% of patients. Signs of increased intracranial pressure were present in 28% of patients, and 9% had cranial nerve symptoms (Table 2).

**Table 2.** Presenting Symptoms

Presenting symptom	*N* (%)	Median survival	95% CI	Sig
Overall survival	1458 (100)	345	326–364	
Focal neurological deficit	935 (64.1)	324	300–348	*P* = .008
Cognitive dysfunction	664 (45.5)	265	245–285	*P* < .001
Headache	517 (35.5)	371	334–407	*P* = .021
Epilepsy	420 (28.8)	409	365–453	*P* < .001
Symptoms of raised ICP^1^	402 (27.6)	316	276–356	ns
Cranial nerve symptom	133 (9.1)	364	304–419	ns

^1^Intra cranial pressure.

### Focal Neurological Deficits

Patients with focal neurological deficits had a significantly shorter OS than patients without focal neurological deficits in univariate analysis (Table 2). Median age was significantly higher (67 years) for patients with focal neurological deficits than for patients without (64 years), *P* = .002. Among those with < 4 cm tumors, there was a survival disadvantage of having focal neurological deficit: OS 341 days (303–379) vs. OS 440 (351–529) for patients without focal neurological deficits, *P* = .001. There was no difference within the other tumor size groups. The difference in survival did not remain in the multivariate analysis (Table 3).

**Table 3.** Multivariate Analysis

	Sig.	Hazard ratio	95.0% CI for hazard ratio	
			Lower	Upper
Age at diagnosis	<0.001	1.024	1.016	1.032
Male (ref)		1.000		
Female	0.758	0.976	0.837	1.138
Tumor size < 4 cm (ref)		1.000		
Tumor size 4–6 cm	0.008	1.266	1.064	1.507
Tumor size > 4 cm	0.020	1.342	1.048	1.719
Biopsy (ref)		1.000		
Partial resection	<0.001	0.509	0.426	0.608
Radical resection	<0.001	0.365	0.297	0.449
WHO performance 0 (ref)		1.000		
WHO performance 1–2	0.616	0.957	0.806	1.136
WHO performance 3–4	0.003	1.493	1.144	1.950
Unmethylated (ref)		1.000		
Methylated	<0.001	0.569	0.481	0.673
RT and chemo (ref)		1.000		
RT only	<0.001	2.436	1.914	3.102
Chemo only	<0.001	2.115	1.666	2.685
Best supportive care	<0.001	3.097	2.421	3.961
No epilepsy (ref)		1.000		
Epilepsy	0.794	1.025	0.849	1.238
No cognitive impairment (ref)		1.000		
Cognitive impairment	0.002	1.283	1.093	1.505
No focal neurological deficit (ref)		1.000		
Focal neurological deficit	0.154	1.128	0.956	1.330
No headache (ref)		1.000		
Headache	0.367	0.925	0.780	1.096

### Cognitive Dysfunction

In univariate analysis, patients with cognitive dysfunction had significantly shorter survival than patients without cognitive dysfunction (Table 2, Figure 1A). Patients with cognitive dysfunction were significantly older (median age 68 years.) than patients without (median age 64 years.) *P* < .001.

The likelihood of having cognitive dysfunction significantly increased with increasing tumor size, from 30% at < 4 cm, to 55% at 4–6 cm, and finally 71% in tumors larger than 6 cm, *P* < .001 comparing all groups. There was a significant difference in survival in patients with tumors < 4 cm; OS was 280 days (240–320) for patients with cognitive dysfunction versus. 424 days (391–457) for patients without cognitive dysfunction, *P* < .001 (Figure 1B). For those in the 4–6 cm tumor group, there was also a significant difference: OS 265 days (237–293) versus 425 days (375–474), *P* < .001. There was no difference in survival for the patients with a > 6 cm tumor, with an OS of 256 days (233–278) for those with cognitive dysfunction versus 251 (172–329) for those without cognitive dysfunction (*P* = .87).

The disadvantage in survival of having cognitive dysfunction was the only symptom significantly related to survival after Cox regression (Table 3). In the treatment naïve model, not including the extent of surgical resection, MGMT promoter methylation status, RT, and CT, the disadvantage in survival of having cognitive dysfunction remained (HR 1.27, 95% CI 1.10–1.46, *P* = .001).

### Headache

Patients who had headaches at presentation survived significantly longer than patients with no headache assessed with univariate analysis (Table 2). Median age was significantly lower among patients with headache than among patients without headache (62 vs. 68 years), *P* < .001. There was a significant difference in survival in the group of patients with 4–6 cm tumors, where patients with headaches survived 392 days (343–440) compared to patients without headaches; OS 307 days (268–345) *P* = .001. Among the smallest and the largest tumors, there were no differences in outcome. The survival advantage did not remain in the Cox regression model (Table 3).

### Epilepsy

Patients presenting with seizures had significantly longer OS in the univariate analysis than patients with no seizures (Table 2, Figure 1C). Patients with epilepsy were significantly younger (median age 64 years.) than patients without seizures (median age 67 years.), *P* < .001. The likelihood of presenting with epilepsy significantly decreased with increasing tumor size, from 45% at < 4 cm to 20% at 4–6 cm and finally 17% in tumors larger than 6 cm, *P* < .001 comparing all groups. Within the group of those having < 4 cm tumors, there was a significant survival benefit for patients presenting with epilepsy, OS 418 days (351–485) versus not presenting with epilepsy OS 326 days (275–377), *P* < .001 (Figure 1D). Within the 4–6 cm group and the > 6 cm group, there were no significant differences in survival.

There was no significant difference in survival in the multivariate model (Table 3).

### Cranial Nerve Symptoms and Symptoms of Raised Intracranial Pressure

There was no difference in survival for patients presenting with cranial nerve symptoms compared to those not having any or when comparing having symptoms of raised intracranial pressure or not.

## Discussion

The findings reveal that initial cognitive dysfunction significantly and negatively impacts both the choice of treatment and the survival rates of glioblastoma patients. This study is, to the best of our knowledge, the largest, to date, to analyze the prognostic implications of presenting symptoms in an unselected glioblastoma patient population. Moreover, it stands out as one of the first studies to utilize real-life, prospectively collected data from a national quality registry for this purpose.^6^ Previous smaller studies have indicated that cognitive impairment and paresis carry a negative prognostic value, while epilepsy at debut has a positive prognostic impact in glioblastoma patients.^11,12,15^ When incorporating known prognostic factors in a multivariate analysis, we found no evidence to support a negative impact of focal neurological deficits nor a positive effect of epilepsy or headache on outcomes.

Presenting symptoms offer readily accessible indicators that can help predict prognosis at the very early stages of the disease. This information is valuable for clinicians, enabling them to discuss life situations and patient prognosis with greater accuracy. We suggest incorporating cognitive dysfunction as a key determinant factor in the development of prognostic models for glioblastoma survival at the group level. This would be similar to the methodology applied in the meningioma model proposed by Zamanipoor Najafabadi et al.^16^

Cognitive dysfunction emerged as one of the most prevalent presenting symptoms in our glioblastoma study, corroborating findings from other research.^12,17^ Identifying these patients can be challenging and they may experience delayed diagnoses, as those with cognitive impairment are often mistakenly diagnosed with depression or other psychiatric disorders.^11,18^

The cause of cognitive dysfunction in glioma patients is believed to be the result of a complex interaction involving tumor volume, volume of surrounding edema, tumor localization, and patient age.^19,20^ Since most cognitive domains rely on widespread cerebral networks, cognitive dysfunction may be considered an indicator of diffuse infiltration.^21^ This study, in line with previous research,^22,23^ has also demonstrated that tumor size possesses prognostic value. Notably, the survival difference between patients with and without cognitive impairment was maintained across subgroups categorized by varying tumor sizes. The negative prognostic value persisted after adjusting for tumor size and other known prognostic factors, including age at diagnosis, sex, PPS, extent of surgical resection, and the use of non-surgical anti-neoplastic treatment.

The reduced overall survival observed in patients with cognitive impairment could be partially attributed to their significantly lower likelihood of receiving radiotherapy and/or chemotherapy. While higher age and larger size of the tumors might also influence this disparity in treatment, further research is needed to conclusively determine their impact. In this context, considering the ethical aspects of refraining from providing patients with cognitive impairment the same treatment as other glioblastoma patients is crucial. It could be argued that patients with cognitive impairments are inadvertently neglected. However, the observed treatment disparities likely result from carefully considered clinical decisions. However, the observed treatment disparities likely result from carefully considered clinical decisions, not to treat patients who might not fully understand their condition, thereby potentially being unable to give informed consent to treatment recommendations. This ethical dilemma underscores the importance of balancing patient autonomy with the need for protective oversight in medical decision-making.^24^ The prioritization of overall quality of life over the pursuit of a marginal survival benefit underscores a compassionate approach to treating frail patients with a limited expected life span. This perspective emphasizes the importance of enhancing the remaining life’s quality rather than extending life at the cost of potentially significant side effects or diminished life quality.

Given the observations that the presence of cognitive dysfunction influences both the extent of surgical resection and the administration of non-surgical anti-neoplastic treatment, we also implemented a treatment naïve model. The results from this model were consistent with those obtained from the other multivariate analysis. This consistency reinforces the impact of cognitive impairment on patient outcomes.

The independent negative prognostic impact of postoperatively cognitive decline on the survival of glioma patients has been recognized for some time, with cognitive deterioration serving as an early indicator of disease progression.^25^ Our study supplies strong support for the notion that cognitive dysfunction serves as a poor prognostic sign from as early as the time of diagnosis. This finding emphasizes the importance of cognitive assessment in the initial evaluation of glioma patients and suggests that cognitive status should be considered when discussing prognosis.

Our study calls into question the previously suggested positive prognostic value of epilepsy at the presentation of the disease.^13,26^ We found that a substantial portion of patients (44%) who presented with seizures had small tumors, measuring less than 4 cm. Within this group with small tumors, there was a significant survival benefit for patients who experienced epilepsy. However, this survival advantage was not observed in patients with larger tumors. Moreover, the survival benefit associated with epilepsy at presentation disappeared after adjusting for known prognostic factors, indicating that the initial observed benefit may be influenced by other variables rather than epilepsy alone. The notably higher likelihood of receiving radical surgery, chemotherapy, and/or radiotherapy could partly explain the survival benefit observed in the univariate analysis. Given these findings, further research is warranted. The previously suggested positive prognostic value of epilepsy may also be attributed to factors such as tumor location. Tumors that trigger seizures are more frequently found in the dominant temporoparietal lobe or subventricular zone.^27^ These locations might have a different impact on patient outcomes compared to tumors in other regions of the brain. Furthermore, the biology of the tumor itself offers a plausible explanation. For instance, glioblastomas presenting with epilepsy have been associated with decreased hypoxia/HIF-1α/STAT5b signaling compared to non-epileptogenic glioblastomas.^28^ This difference in cellular signaling could contribute to the variations in patient survival.

In newly diagnosed glioma patients issues such as fatigue and motor symptoms are common and negatively affect aspects of the patient’s functioning and quality of life although they do not impact survival.^17^ In our study, univariate analysis initially indicated that patients with focal neurological deficits, such as motor symptoms had shorter survival while patients with headaches experienced longer survival. However, these differences were not maintained in the multivariate analysis, suggesting that when other prognostic factors are considered the initial survival association was not directly related to the symptoms themselves.

### Strengths and Limitations of This Study

The strength of this study lies in the size of its cohort, with 1458 glioblastoma patients included. This represents nearly the entire glioblastoma cohort in Sweden during the study period. To the best of our knowledge, this is the largest study of its kind and stands out as one of the few studies utilizing this approach. It features a prospective sampling of real-life clinical data, and the validity of recorded variables when compared to medical reports, is generally good. This robust dataset provides a strong foundation for the study´s findings.

The study, as with all register-based research, faces limitations related to its data collection methodology. The data were not collected directly by the research group but by clinicians across Sweden, which could impact the quality and consistency of the collected data. Additionally, there is a noted delay in reporting non-surgical anti-neoplastic treatment to the registry. In about 27% of cases, reporting was incomplete. Consequently, this limitation meant, that the multivariate model could only include 1068 patients. We suspect that best-supportive care is overrepresented amongst the missing data since the motivation to register might be higher when the patients receive active oncological treatment. This shortfall highlights the challenges of relying on registry data for comprehensive analysis and underscores the importance of accurate and timely data reporting in healthcare settings. However, when we excluded surgical and non-surgical treatment in our treatment naïve multivariate model, the negative effect of cognitive impairment was still significant.

Additionally, in real life, not all patients with suspected glioblastoma are eligible for surgery and only those with histologically verified glioblastoma were included.

## Conclusion

This extensive real-life study reveals that initial cognitive impairment acts as an independent negative predictive factor for treatment decisions and adversely affects survival outcomes in glioblastoma patients.

## Data Availability

The data will be made available from the Swedish Brain Tumour registry upon reasonable request.
